# Long-Range GABAergic Projections of Cortical Origin in Brain Function

**DOI:** 10.3389/fnsys.2022.841869

**Published:** 2022-03-22

**Authors:** Jocelyn Urrutia-Piñones, Camila Morales-Moraga, Nicole Sanguinetti-González, Angelica P. Escobar, Chiayu Q. Chiu

**Affiliations:** ^1^Ph.D. Program in Neuroscience, Facultad de Ciencias, Universidad de Valparaíso, Valparaíso, Chile; ^2^Facultad de Ciencias, Instituto de Neurociencia, Universidad de Valparaíso, Valparaíso, Chile; ^3^Centro Interdisciplinario de Neurociencia de Valparaíso, Universidad de Valparaíso, Valparaíso, Chile; ^4^Instituto de Fisiología, Facultad de Ciencias, Universidad de Valparaíso, Valparaíso, Chile; ^5^Centro de Neurobiología y Fisiopatología Integrativa, Universidad de Valparaíso, Valparaíso, Chile

**Keywords:** long-range GABAergic axons, inhibitory connections, disinhibition, hippocampus, neocortex, cortico-cortical, corticofugal

## Abstract

The study of long-range GABAergic projections has traditionally been focused on those with subcortical origin. In the last few years, cortical GABAergic neurons have been shown to not only mediate local inhibition, but also extend long-range axons to remote cortical and subcortical areas. In this review, we delineate the different types of long-range GABAergic neurons (LRGNs) that have been reported to arise from the hippocampus and neocortex, paying attention to the anatomical and functional circuits they form to understand their role in behavior. Although cortical LRGNs are similar to their interneuron and subcortical counterparts, they comprise distinct populations that show specific patterns of cortico-cortical and cortico-fugal connectivity. Functionally, cortical LRGNs likely induce timed disinhibition in target regions to synchronize network activity. Thus, LRGNs are emerging as a new element of cortical output, acting in concert with long-range excitatory projections to shape brain function in health and disease.

## Introduction

Cortical inhibitory GABAergic neurons can act locally or remotely through short-range or long-range axons, respectively (Tamamaki and Tomioka, 2010; Caputi et al., 2013). They have been studied mainly with respect to their local synaptic interactions within the cortical microcircuitry (DeFelipe, 2002; Markram et al., 2004; Monyer and Markram, 2004). Although they represent only a small percentage of cortical GABAergic cells in rodents, cats and monkeys (Gonchar et al., 1995; Tomioka et al., 2005; Higo et al., 2007; Tomioka and Rockland, 2007), long-range GABAergic neurons (LRGNs; see list of abbreviations in Table 1) are emerging as key players in cortical function (Lee et al., 2014; Basu et al., 2016; Melzer et al., 2017).

**TABLE 1:** Abbreviations and acronyms used in this review.

Abbreviation/Acronym	Expression
AC	Auditory cortex
AC-LA LRGNs	AC LRGNs projecting to LA
ACh	Acetylcholine
BLA	Basolateral amygdala
CA1-MEC LRGNs	CA1 LRGNs projecting to MEC
CA1-RSC LRGNs	CA1 LRGNs projecting to RSC
CeA	Central nucleus of the amygdala
CCK	Cholecystokinin
D1R-MSNs	MSNs expressing dopamine receptor type 1
D2R-MSNs	MSNs expressing dopamine receptor type 2
DG	Dentate gyrus
DG-MS LRGNs	DG LRGNs projecting to the MS
DR1	Dopamine receptor type 1
DR2	Dopamine receptor type 2
EC	Entorhinal cortex
eCBs	endocannabinoids
GC	Granule cells
GPi	Internal globus pallidus
HDB	Horizontal diagonal band of Broca
HDB-OB LRGNs	HDB LRGNs projecting to the OB
HILs	Hilus associated interneurons
HPC	Hippocampus
INs	GABAergic interneurons
LA	Lateral amygdala
LEC	Lateral entorhinal cortex
LEC-CA1 LRGNs	LEC LRGNs projecting to CA1
LH	Lateral hypothalamus
LRGNs	Long-range GABAergic neurons
M1	Primary motor cortex
M2	Secondary motor cortex
MC	Motor cortex
MC-SC LRGNs	MC LRGNs projecting to SC
MEC	Medial entorhinal cortex
mPFC	Medial prefrontal cortex
mPFC-NAcc LRGNs	mPFC LRGNs projecting to the NAcc
MS	Medial septum
MSNs	Medium spiny neurons
NAcc	Nucleus Accumbens
nNOS	Neuronal nitric oxide synthase
NPY	Neuropeptide Y
OB	Olfactory bulb
PFC-HPC LRGN	PFC LRGNs projecting to the HPC
PFC-NAcc LRGNs	PFC LRGNs projecting to the NAcc
PV	Parvalbumin
PV-LRGNs	LRGNs positive for PV
PV-M1-LRGNs	M1-LRGNs positive for PV
PV-M2-LRGNs	M2-LRGNs positive for PV
Reelin/NPY-LRGNs	LRGNs positive for Reelin or NPY
RSC	Retrosplenial cortex
SC	Somatosensory cortex
SC-MC LRGNs	SC LRGNs projecting to MC
SLM	Stratum lacunosum moleculare
SNr	Substantia nigra pars reticulata
SO	Stratum oriens
SOM	Somatostatin
SOM-LRGNs	LRGNs positive for SOM
SOM-M1–LRGNs	M1-LRGNs positive for SOM
SOM-M2–LRGNs	M2-LRGNs positive for SOM
SR	Stratum radiatum
VIP	Vasoactive intestinal peptide
VTA	Ventral tegmental area
VTA-DG	VTA projections to DG
VTA–HPC-LRGNs	VTA LRGNs projecting to HPC
5-HT3aR	Ionotropic serotonin receptor 5-HT3A

Thus far, most of the work has focused on characterizing LRGNs, with a few papers having studied their impact on neural circuit function. In this review, we will describe the diverse anatomical circuits formed by LRGNs across different cortical regions. Whenever possible we will highlight the pre and postsynaptic cell types that compose the circuit, incorporating their morphological and functional characteristics. As a comparison, we will briefly introduce essential features of local inhibitory circuits in the cortex as well as classical non-cortical GABAergic projections. Cortical LRGNs are defined as GABA-producing neurons that extend their axons outside of their region of origin, e.g., the hippocampus (HPC) or neocortex, enabling communication between functionally distinct brain regions (Tamamaki and Tomioka, 2010; Caputi et al., 2013; Melzer and Monyer, 2020). According to this definition, cortical LRGNs comprise a heterogeneous population, showing differences in morphology, intrinsic membrane properties and expression of molecular markers. Despite such diversity, cortical LRGNs have in common the propensity to target GABAergic neurons (Toth et al., 1997; Melzer et al., 2012; Caputi et al., 2013; Melzer and Monyer, 2020), thereby mainly mediating long-range disinhibition. The impact of such GABA-GABA cell connectivity depends on the precise cellular subtypes targeted and the microcircuits in which they participate, adding to the complexity of circuit mechanisms that regulate brain function.

To understand how cortical LRGNs uniquely contribute to brain function and behavior, we will start by introducing key features of their better-known counterparts, namely cortical GABAergic interneurons (INs) and subcortical GABAergic projection neurons. Cortical LRGNs will be discussed depending on whether they originate from the HPC or the neocortex, two regions of intense research. Finally, we will summarize works that reveal LRGN modulation of behaviors and postulate general principles underlying LRGN function.

## Properties and Characteristic of Interneurons

GABA-producing neurons constitute the primary cellular population that mediates inhibition in the cortex. Their postsynaptic targets are typically local neurons; thus, these GABAergic cells are commonly referred to as INs (DeFelipe, 2002; Markram et al., 2004; Monyer and Markram, 2004). Cortical GABAergic INs are highly diverse, identified by a range of neurochemical markers, firing patterns, morphological features and subcellular targets (Miles et al., 1996; Gonchar and Burkhalter, 1997; Petilla Interneuron Nomenclature Group [PING], Ascoli et al., 2008; Xu et al., 2010; Rudy et al., 2011; Tremblay et al., 2016). A popular GABAergic IN classification scheme is based on the non-overlapping co-expression of either parvalbumin (PV), somatostatin (SOM), or the ionotropic serotonin receptor 5-HT3A (5-HT3aR). PV-INs comprise ∼40% of cortical GABAergic neurons and target peri-somatic and axon initial segment subregions of pyramidal neurons to control their spiking output (Kvitsiani et al., 2013; Hu et al., 2014). SOM-INs make up ∼30% of cortical GABAergic neurons and mostly form synapses onto distal dendrites of principal neurons to regulate excitatory synaptic signaling (Wang et al., 2004; Murayama et al., 2009; Chiu et al., 2013). The remaining 5HT3aR-INs are less understood, consisting of a diverse group of mostly dendrite-targeting cells that includes INs expressing the vasoactive intestinal peptide (VIP) and cholecystokinin (CCK; Rudy et al., 2011; Tremblay et al., 2016). It is important to note each of these IN categories contains heterogeneous subpopulations of cells and ongoing efforts currently aim to classify them more precisely. Recent single-cell RNA sequencing studies reveal IN clusters that often correspond to previously defined cell types (Fishell and Rudy, 2011; Zeisel et al., 2015; Tasic et al., 2018), which can be further subdivided depending on a combination of morphological, electrophysiological and transcriptomic features into “met-types” (Gouwens et al., 2020). It remains unclear whether these transcriptionally defined met-types reflect core cellular entities or map onto a continuum of developmental and activity-dependent states.

At the cellular level, specialized GABAergic IN subtypes fine-tune the functional integration of input signals. Whereas PV-INs mediate feedforward inhibition, SOM-INs are responsible for feedback inhibition (Pouille and Scanziani, 2001; Kapfer et al., 2007; Silberberg and Markram, 2007). Although VIP-INs make inhibitory synapses on pyramidal neurons, they are thought to primarily inhibit other GABAergic cells to drive disinhibition of the cortex and HPC (Acsady et al., 1996; Lee et al., 2013; Pi et al., 2013). Notably, the different IN classes can be reciprocally interconnected (Cottam et al., 2013; Pfeffer et al., 2013), enabling bidirectional communication that adds to the computational power of microcircuit processing. The activity of specific GABAergic IN subtypes has been linked to distinct temporal patterns in the local circuit (Klausberger and Somogyi, 2008; Cardin, 2018), reflecting differential interactions of these INs with local excitatory networks (Caroni, 2015). For instance, PV-INs in the neocortex may entrain rhythmic activity in the gamma-range (Cardin et al., 2009; Sohal et al., 2009) to facilitate sensory and cognitive processing. In HPC, PV-INs fire at different time-points of a theta-oscillatory cycle and in spatial exploration tasks compared to those that express CCK (Freund, 2003; Klausberger et al., 2005), suggesting that even basket cells can be subdivided to support different aspects of exploratory behavior. As cortical LRGNs also express these neurochemical markers of GABAergic INs, it will be crucial to determine the extent to which the well-characterized properties of inhibitory neurons apply in the context of long-range inhibition.

## Subcortical Long-Range GABAergic Neurons

The existence of long-range GABAergic projections in the brain has long been appreciated, especially from subcortical regions that comprise a substantial population of GABAergic neurons such as the striatum, amygdalar complex and brainstem. Although Purkinje cells of the cerebellum are classical long-range GABAergic projecting neurons, they have not been shown to be innervated by cortical LRGNs thus far and therefore are not included in this review. Subcortical GABAergic projections are briefly discussed here with the aim of contrasting their characteristics with the more recently appreciated cortical counterparts as well as to provide background to understand the potential impact of long-range cortical inhibition onto these regions. We refer to excellent reviews on the organization and function of subcortical GABAergic projections elsewhere (Calabresi et al., 2014; Gordon-Fennell and Stuber, 2021).

The striatum works as an integrating hub, where principal medium spiny neurons (MSNs) receive glutamatergic inputs from different cortical and thalamic areas, and in the case of the ventral portion, also from the ventral HPC and amygdala (Berendse and Groenewegen, 1990; Wall et al., 2013; Nelson and Kreitzer, 2014; Mandelbaum et al., 2019). Furthermore, the excitatory activity of MSNs is locally regulated by GABAergic and cholinergic INs (Tepper et al., 2008) and differentially modulated by dopaminergic inputs from the substantia nigra pars compacta and the ventral tegmental area (VTA) depending on the postsynaptic expression of type 1 (D1R-MSN) or type 2 (D2R-MSN) dopamine receptors (Le Moine and Bloch, 1995; Gerfen and Surmeier, 2011; Tritsch and Sabatini, 2012). MSNs send profuse GABAergic projections to neighboring basal ganglia nuclei (Kemp and Powell, 1971; Smith et al., 1998; Tepper et al., 2007) and have been extensively studied to understand their contribution to the generation of motor learning, habitual and motivated behaviors, mood and reward (Gerfen and Surmeier, 2011; Tritsch and Sabatini, 2012; Bariselli et al., 2019). By inhibiting the main GABAergic output nuclei of the basal ganglia to the thalamus (thalamic disinhibition), D1R-MSNs of the direct pathway facilitate motor behavior or the approach to a rewarding stimulus. In contrast, D2R-MSNs of the indirect pathway, acting through multiple stages within the basal ganglia, produce inhibition of the thalamus to suppress movements or to avoid non-rewarding stimuli (Beckstead and Cruz, 1986; Alexander and Crutcher, 1990; Hedreen and DeLong, 1991). Interestingly, the basal ganglia may also exert its effects independent of the thalamus as it also sends GABAergic projections directly to the neocortex (Jayaraman, 1980; Saunders et al., 2015; Sun Q. et al., 2019). In a mapping study using monosynaptic viral tracers, these particular long-range GABA neurons have been shown to target GABAergic INs (Sun Q. et al., 2019), indicating that the basal ganglia may control their own cortical feedback *via* disinhibition.

The amygdala is made up of a diverse collection of interconnected nuclei to regulate emotionally relevant behaviors (LeDoux, 2007; Janak and Tye, 2015). For example, the central nucleus of the amygdala (CeA) is a striatal-like GABAergic structure that projects to the hypothalamus and brainstem to initiate fear responses (Pare and Duvarci, 2012). Recent viral tracing work suggests that single neurons in the CeA send bifurcating projections to the medial prefrontal cortex (mPFC) and the ventrolateral periaqueductal gray (Sun Y. et al., 2019) to potentially dually regulate adaptive emotional responses. Interestingly, such GABAergic projections were also found to originate from the basolateral amygdala (BLA), a region thought to be cortical-like and predominately populated by glutamatergic neurons. This finding adds to the growing evidence that GABAergic cells in the BLA are not solely INs but can also send long-range axons to distal regions like the entorhinal cortex (EC; McDonald and Zaric, 2015) and basal forebrain (McDonald et al., 2012). Indeed, the entire amygdaloid complex may make GABAergic connections throughout the brain. Large intercalated cells of the amygdala, many of which are PV+, project to the perirhinal, entorhinal, and piriform cortex, synapsing selectively on GABAergic cells (Bienvenu et al., 2015). Furthermore, neurons in the bed nucleus of the stria terminalis, a heterogeneous extension of the amygdala, provide distal GABAergic inputs onto non-dopaminergic, likely GABAergic (Nair-Roberts et al., 2008), neurons in the VTA to promote active reward seeking (Jennings et al., 2013). Indeed, GABAergic INs in the VTA are preferred targets of inhibitory inputs from numerous subcortical regions, including the lateral hypothalamus (Nieh et al., 2015, 2016), medial preoptic area (McHenry et al., 2017) and the nucleus accumbens (NAcc; Bocklisch et al., 2013). A recent viral genetic mapping study revealed that whereas GABA INs in the VTA strongly connect to nearby dopaminergic neurons, distal GABAergic inputs preferentially target local GABA INs (Soden et al., 2020). Given that GABA cells in the VTA also receive excitatory inputs from diverse brain areas (Morales and Margolis, 2017; Beier et al., 2019; Bouarab et al., 2019), they likely act as key integrators of information about the external environment and internal state to control dopaminergic neuromodulation in motivated behavior.

In addition to mediating local inhibition, GABA neurons in the VTA also provide long-distance inhibition to several distinct brain regions, including the basal ganglia, amygdala, dorsal raphe nucleus, HPC and prefrontal cortex as revealed by retrograde tracing (Ntamati and Luscher, 2016; Bouarab et al., 2019; Breton et al., 2019). Interestingly, discrete subsets of these neurons target different brain structures, with little axonal collateralization, suggesting independent parallel circuits. Because they have mostly been identified anatomically and not characterized functionally (but see the section “Neurotransmitter Co-release in Local and Distant Inhibition”), how the distinct GABAergic projections from VTA subregions act in parallel to regulate different facets of reward and aversion remains to be determined.

Besides its better known endocrine function, the hypothalamus, at least the lateral portion (LH), is another important brain structure that sends GABAergic projections to distant regions like the basal forebrain (Cassidy et al., 2019), VTA (Nieh et al., 2015, 2016), and dorsal pons (Marino et al., 2020) to regulate motivated behaviors like feeding and social approach. Interestingly, optogenetic activation of inhibitory and excitatory inputs from LH to the VTA disinhibited and suppressed dopamine neurons, respectively, presumably with local VTA INs as common postsynaptic targets, thereby exerting opposing influences on social approach and interaction (Nieh et al., 2016). Studies employing similar strategies suggest that the brain circuitry underlying compulsive overeating may involve GABA-GABA connectivity between LH and a region medial to the locus coeruleus but not between LH and VTA (Marino et al., 2020). Taken together, GABA is a major neurotransmitter in subcortical long-range communication, often operating *via* disinhibition in downstream brain regions.

## Cortical Long-Range GABAergic Neurons

An essential characteristic of cortical areas is its organization in layers, which is thought to be crucial for amplifying and computing incoming signals. Here, we will consider as cortical structures the neocortex and the HPC (sometimes referred to as allocortex). Early evidence suggesting the existence of cortical LRGNs came from the observation of non-pyramidal retrogradely labeled somata in the cortex (Code and Winer, 1985; Totterdell and Hayes, 1987; Hughes and Peters, 1990). The advent of improved viral tracing techniques in transgenic mice to selectively monitor and manipulate GABAergic neurons enabled more precise interrogation of LRGN connectivity and functional impact.

**FIGURE 1:**
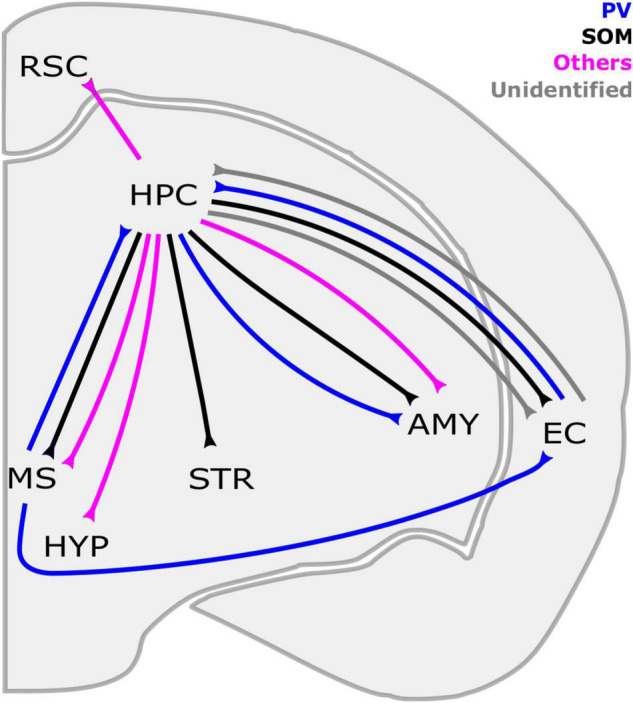
Long-range GABAergic neuron (LRGN) projections to and from the hippocampus. The HPC is reciprocally connected with the EC and MS through SOM-LRGNs (HPC outputs) and PV-LRGNs (HPC inputs). Even a single LRGN in the MS can project to both the EC and HPC. In parallel, LRGNs that are identified by markers other than PV and SOM (magenta), such as Reelin/NPY, in the HPC send axons to the RSC. Inhibitory projections from the HPC to subcortical structures have been described, such as the STR and the AMY. HPC-AMY LRGNs are diverse in their neurochemical expression, including SOM, PV and others. The HPC also sends nNOS + LRGNs to the HYP and the MS. Contralateral projections are not included here. AMY, amygdala; EC, entorhinal cortex; HYP, hypothalamus; HPC, hippocampus; MS, medial septum; RSC, retrosplenial cortex; STR, striatum; PV, parvalbumin, SOM, somatostatin; NPY: neuropeptide Y; nNOS, neuronal nitric oxide synthase.

In the context of cortical LRGNs, those originating from HPC are among the best described. As the HPC is well known for coordinating spatial and contextual information *via* its various subfields (Yassa and Stark, 2011; Lee et al., 2020), we will describe key features of LRGNs located in different subregions of the hippocampal circuit highlighting their connectivity with distal structures such as the medial septum (MS) and EC (Figure 1 and Table 2) that are important for navigation and contextual learning. For simplicity, hippocampal GABAergic innervations in remote subcortical regions such as the NAcc (Totterdell and Hayes, 1987) are not discussed below.

**TABLE 2:** Cortical long-range GABAergic neurons (LRGNs).

From	To	Markers	Known characteristics	References
**Hippocampal LRGNs**				
HPC	MEC	SOM	LRGNs in DG and CA1 preferentially inhibit GABAergic neurons in MEC and to a lesser extent inhibit stellate cells.	Ino et al., 1990; Melzer et al., 2012
	MS	SOM	Present in all hippocampal subfields (DG, CA1, CA3). LRGNs in the hilus of the DG inhibit glutamatergic, cholinergic and GABAergic neurons of the MS. Also these neurons inhibit local PV-INs in DG through axon collaterals. CA3 and CA1-LRGNs co-express SOM as well as other molecular markers.	Jinno et al., 2007; Yuan et al., 2017
	AMY	PV and SOM	LRGNs comprise 17% of the GABAergic neurons in the ventral hippocampus.	Ino et al., 1990; Lubkemann et al., 2015
	STR	SOM	Postsynaptic targets and their function remains unknown.	Melzer et al., 2012
	Extra-hippocampal areas	nNOS	These LRGNs project to dorsal subiculum, entorhinal cortex, mammillary nuclei, lateral hypothalamus, olfactory tubercle, olfactory bulb, ipsilateral dentate gyrus, tenia tecta, medial septum (co express SOM) diagonal band of Broca. Also to the contralateral hippocampal formation. Additionally, they project locally to CA1, showing co-expression of NPY, SOM, CR, VIP, or muscarinic receptor 2 (M2).	Christenson Wick et al., 2019
	RSC	Reelin/NPY	LRGNs located in CA1 establish synapses in layer V pyramidal neurons.	Jinno et al., 2007; Miyashita and Rockland, 2007; Yamawaki et al., 2019
**Cortifugal LRGNs**				
SC	Caudal STR	PV	They represent 3% of cortico-striatal neurons	Jinno and Kosaka, 2004
RSC	STR	PV	They represent 5% of cortico-striatal neurons	Jinno and Kosaka, 2004
MC	STR*	PV, SOM, and VIP	-PV-M1-LRGNs mainly inhibit D1R-MSNs and decrease locomotion. They also target D2R-MSNs and cholinergic interneurons. -SOM-M1-LRGNs primarily inhibit cholinergic INs and increase locomotion. Additionally, they target D2R and D1R MSNs. -SOM-M2-LRGNs mainly inhibit striatal D1R-MSN and decrease locomotion. They also target D2R-MSNs and to a lesser extent cholinergic interneurons. -VIP-M1-LRGNs innervate the striatum.	Rock et al., 2016; Melzer et al., 2017; Bertero et al., 2021
AC	STR	PV, SOM, and VIP	SOM-LRGNs inhibit MSN in dorsal striatum. PV and VIP-LRGNs project to striatum.	Rock et al., 2016; Bertero et al., 2019; Bertero et al., 2020
	AMY	SOM and VIP	SOM-LRGNs inhibit principal neuron in lateral amygdala. VIP-LRGNs project to amygdala, medial geniculate body and superior and inferior colliculi.	Bertero et al., 2019; Bertero et al., 2021
mPFC	STR and AMY	PV and VIP	mPFC-LRGNs inhibit MSNs in ventral striatum and elicit avoidance. Also, mPFC-LRGNs project dorsal striatum, amygdala and claustrum.	Lee et al., 2014
OFC	Several subcortical areas	SOM and PV	Wide projections to caudate putamen (PV, NPY or SOM), ventral pallidum, lateral globus pallidus, nucleus accumbens, and olfactory tubercle. Minor projections to mediodorsal thalamic nucleus (negative for: PV, SOM, VIP, NPY, and nNOS), diagonal band of Broca, medial globus pallidus, substantial nigra, and dorsal raphe nucleus (expressing PV, the expression of other markers was not tested). Some contralateral subcortical projections were also observed, for more detail, see Tomioka et al. (2015).	Tomioka et al., 2015
**Neocorti-cortical LRGNs**				
SC	Ipsilateral VC and MC	SOM	Additionally some SC-LRGNs express nNOS and NPY, likely co-expressing one of this markers with SOM.	Tomioka et al., 2005
	Contralateral SC	–	The specific subtypes need to be determined (rat).	Gonchar et al., 1995
MC	Ipsilateral VC, SC, and AC*	SOM	SOM-LRGNs also project to mPFC, RSC and other structures*. Additionally, some MC-LRGNs express nNOS and NPY, likely co-expressing one of this markers with SOM. Postsynaptic targets and their function remains undetermined.	Tomioka et al., 2005; Melzer et al., 2017
	Contralateral MC	PV, VIP	Axons of PV-LRGNs cross through the CC and reach the contralateral MC. They show basket morphology and similar electrophysiological properties compared to PV-INs. Also VIP-LRGNs project to contralateral MC.	Rock et al., 2018; Bertero et al., 2021
AC	Ipsilateral VC and SC	VIP	VIP-LRGNs project ipsilaterally to the temporal association cortex.	Bertero et al., 2021
	Contralateral AC	PV and VIP	Basket cell-looking PV-LRGNs project to contralateral hemisphere through CC. They receive thalamic afferents and innervate local pyramidal neurons. Electrophysiological properties of layer V PV-LRGNS and PV-INs are different. Additionally, VIP-LRGNs project to contralateral AC.	Rock et al., 2018; Zurita et al., 2018; Bertero et al., 2021
VC	Contralateral VC	PV	As in AC, PV-LRGNs have basket morphology and similar electrophysiological properties compared to PV-INs. Their axons cross through the CC and reach the contralateral VC.	Rock et al., 2018
mPFC	Ipsilateral MC	SOM	SOM-LRGNs project ipsilaterally to MC.	Tomioka et al., 2005
OFC	Ipsilateral	–	Project to insular, motor, lateral entorhinal, peri-rhinal, and somatosensory cortex.	Tomioka et al., 2015
	contralateral	–	Project contralaterally to granular insular, infralimbic, and orbitofrontal cortices.	Tomioka et al., 2015
EC	HIP	PV	MEC-LRGNs inhibit GABAergic neurons. LEC-LRGNs inhibit CCK-INs and their terminals seem to integrate multisensory signals. Additionally, the molecular identity of some of the MEC-HPC LRGNs has not been identified.	Melzer et al., 2012; Basu et al., 2016

Summary table of hippocampal, cortifugal, and cortico-cortical LRGNs showing their region of origin (from), target regions (to), molecular identity, if known (markers), important characteristics such as their cellular targets (known characteristics) and the references of the corresponding works (primarily carried out in mice and to a lesser extent in rats). *Melzer et al. (2017), also showed anatomical evidence of SOM-LRGNs in M1 and M2 projecting to other cortical and subcortical regions [for more details review supplementary data of Melzer et al. (2017)].

### Hippocampal Long-Range GABAergic Neurons

#### Dentate Gyrus

Primary excitatory inputs to the HPC arrive in the molecular layer of the dentate gyrus (DG) from layer II/III of EC, a region with medial (MEC) and lateral (LEC) anatomical subdivisions (Amaral and Witter, 1989; Witter et al., 2017). These glutamatergic perforant paths depolarize the dendrites of principal granule cells (GC), whose soma are in the granular layer. In parallel, several hilar SOM + GABAergic cells mediate local inhibition as hilar-perforant path associated interneurons (HIPPs; Bakst et al., 1986; Halasy and Somogyi, 1993; Han et al., 1993) to modulate GC dendritic excitability. Notably, SOM + neurons in the hilus also send feedback inhibitory projections to the MEC as revealed in retrograde tracer studies with *post-hoc* immunohistochemistry (Ino et al., 1990; Melzer et al., 2012) to potentially control incoming perforant path activity. GABAergic cells in the hilus have also been shown to project to other distal regions such as the subiculum and MS (Jinno and Kosaka, 2002; Jinno et al., 2007). More recently, a new subclass of SOM + septal-projecting LRGNs in the hilus, called hilus-associated INs (HILs), has been shown to also send local axon collaterals to exert strong perisomatic inhibition over DG PV-INs (Yuan et al., 2017), and thus disinhibiting GCs and facilitating GC-CA3 communication. The impact of these HILs in setting MS activity is unclear, as they functionally target PV + GABAergic, cholinergic and glutamatergic neurons, as confirmed by optogenetic stimulation and *post-hoc* immunohistochemistry. Regardless, HILs may powerfully determine the output of the target cell in MS by establishing perisomatic inhibition as in the DG, unlike typical cortical SOM-INs. Given demonstrated reciprocal LRGN interconnectivity between the HPC and MS (Toth et al., 1993; Jinno et al., 2007; Takacs et al., 2008), HILs may also be reciprocally inhibited by these septal LRGNs. This possibility remains to be investigated. Moreover, individual GABAergic LRGNs in the MS can send projections to both the HPC and MEC, making synaptic contacts with different IN subtypes (Fuchs et al., 2016). These dually projecting LRGNs may help to coordinate the activity of these three areas: HPC, MS, and EC.

#### Cornu Ammonis Subareas

CA3 is the second stage of the trisynaptic circuit of the HPC and receives excitatory axon fibers from GCs of the DG (called mossy fibers) and direct glutamatergic inputs from layer II of the EC in *stratum lacunosum moleculare* (SLM). CA3 pyramidal neurons project their excitatory axons through Schaffer collaterals in the *stratum radiatum* (SR) to the CA1 area. CA1 pyramidal cells are the main output of the HPC, but also receive direct excitatory inputs from the EC (Amaral and Witter, 1989). Additionally, pyramidal cells interact with a plethora of different INs subtypes distributed across all layers of the CA region (Klausberger and Somogyi, 2008).

##### CA3

Although distributed LRGNs in CA3 have been found, not much information about them is available. A common feature of CA3-originating LRGNs is that they express SOM as a molecular marker but are heterogeneous, showing a diversity of co-expression with other markers (Zappone and Sloviter, 2001; Jinno and Kosaka, 2002; Jinno et al., 2007). The soma of MS-projecting LRGNs are located in all CA3 sublayers whereas those of LRGNs that project to the subiculum are restricted to SR/SLM (Jinno et al., 2007). The function of hippocampal GABAergic projections from CA3 is unknown. Notably, GABAergic cells in the CA3 have been shown to receive long-range inhibitory projections from PV + LRGNs in the MEC (Melzer et al., 2012) and MS (Freund and Antal, 1988). Combining anterograde labeling with immunohistochemistry, it was shown that GABAergic septo-hippocampal projections target SOM-, VIP- and CCK-INs in CA3 as well as other areas of the HPC (Gulyas et al., 1990). Stimulation of the septo-hippocampal connections triggers disinhibition of CA3 pyramidal cells (Toth et al., 1997) and alters network activity (Joshi et al., 2017; Unal et al., 2018; Salib et al., 2019). This may also apply for the entorhinal-hippocampal inhibitory afferents. For a better understanding of the CA3 long-range inhibitory connectivity, further research is needed.

##### CA1

As previously mentioned, CA1 serves as the final stage of the trisynaptic HPC circuit, moving highly processed information back to the EC, with a synaptic relay in the subiculum. In contrast to CA3, CA1 LRGNs that send axons to the MEC are located in the *stratum oriens* (SO) and SR, extending hundreds of microns horizontally throughout MEC layer I (Melzer et al., 2012). Whereas the targets of these long-range projections are a heterogeneous population of GABAergic cells identified electrophysiologically, CA1-MEC LRGNs are most frequently characterized by SOM expression (Jinno et al., 2007) as are a primary IN population in the SO that sends axons to the SLM (called O-LM cells). It remains to be determined whether O-LM INs may also be LRGNs in CA1. Moreover, GABAergic cells in SO of CA1 also project to the MS, but given the lack of tracer co-labeling, these cells are likely distinct from those projecting to the MEC. Because these CA1-MS GABAergic cells synapse onto local INs and remote MS GABA neurons, they likely serve double duty, simultaneously disinhibiting CA1 and MS (Gulyas et al., 2003). Interestingly, the dorsal and ventral HPC are also connected by these cells (Gulyas et al., 2003), potentially coordinating activity between the two portions of the HPC that play differential roles in memory and emotional processes, respectively (Fanselow and Dong, 2010).

Recently, using an intersectional viral transduction approach, GABAergic cells in the SO and *stratum pyramidal* (SP) have been discovered to also project to extrahippocampal regions such as the band of broca and the tenia tectum in the frontal cortex (Christenson Wick et al., 2019). Notably, these cells typically express the neuronal nitric oxide synthase (nNOS) and neuropeptide Y (NPY) but not other IN markers such as PV, SOM or VIP. Moreover, these nNOS + LRGNs also form GABAergic synapses onto local pyramidal neurons and INs in CA1, exhibiting similar inhibitory postsynaptic response characteristics irrespective of target cell type. Optogenetic activation of these nNOS + cells increases local field potential (LFP) power at the stimulated frequency within the HPC and frontal cortex individually as well as enhances LFP coherence between these two regions, suggesting that they coordinate both local network activity and inter-regional communication.

GABAergic cells in CA1 have been shown to project to the retrosplenial cortex (RSC; Jinno et al., 2007; Miyashita and Rockland, 2007), another cortical structure that is part of the navigation system. In contrast to the CA1-MEC LRGNs, these CA1-RSC LRGNs are principally located at the SR/SLM border and make functional synapses onto the apical dendritic tufts of layer V pyramidal neurons, to potentially modulate synaptic excitation from the anterior thalamic nuclei (Yamawaki et al., 2019). Interestingly, the SR/SLM border of CA1 appears to be a site of integration, receiving long-range inhibitory PV + axons from MEC and LEC (Melzer et al., 2012; Basu et al., 2016) and excitatory perforant path inputs from the EC. Indeed, LRGNs from LEC have been reported to target CCK + INs to facilitate the integration of perforant path and CA3 inputs in CA1 pyramidal neurons (Basu et al., 2016). In addition, CCK-INs located in SP and SR, as well as SOM-INs in SO, receive GABAergic projections from the MS (Gulyas et al., 1990), which are probably PV + (Freund and Antal, 1988). In brain slice electrophysiological recordings, synaptic terminals of PV + septal-hippocampal LRGNs display a lower probability of GABA release and reduced short-term synaptic depression that is frequency-dependent compared to that of hippocampal PV-INs (Yi et al., 2021), suggesting differential activity-dependent recruitment of local vs. distal inhibition in the HPC.

The HPC is a coordination center for several cortical and subcortical areas to drive behaviors such as navigation, learning and memory. Like glutamatergic connections, LRGNs also form a large interconnected inhibitory network linking HPC, EC, and MS. Given the importance of the MS in theta oscillations in MEC and HPC and spatial navigation (Mitchell et al., 1982; Hagan et al., 1988; McNaughton et al., 2006), particularly the GABAergic constituency (Dwyer et al., 2007; Pang et al., 2011), LRGNs from and to the HPC may contribute to shape such rhythmic activity and modulate exploratory behavior.

### Neocortical Long-Range GABAergic Neurons

**FIGURE 2:**
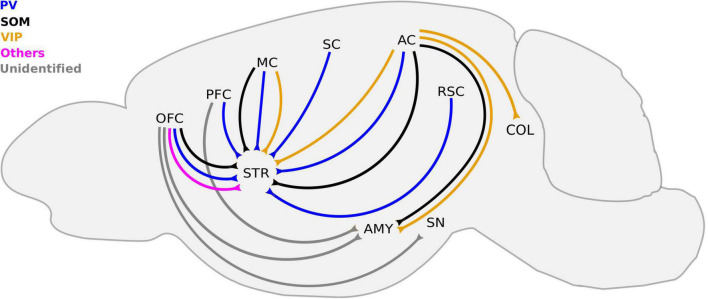
Known cortico-fugal LRGNs. LRGNs in the neocortex project to a variety subcortical areas, some of which are not included here. For simplicity, we highlight those that have been functionally characterized or investigated in more detail. Corticofugal LRGNs are diverse in their molecular markers. The PFC sends PV-LRGN projections to the STR, and the molecular identity of LRGNs to the AMY remains unidentified. SOM+, PV+, and other LRGNs (magenta) in the OFC send axons to the STR, and those that project to the AMY and SN are unidentified (gray). SOM+, PV+, and VIP+ axons to the STR arise from the MC and AC, which also sends SOM+ and VIP+ projections to the AMY and COL. The SC and RSC contain PV-LRGNs that project to the STR. AC, auditory cortex; AMY, amygdala; COL, colliculus; MC, motor cortex; OFC, orbitofrontal cortex; PFC, prefrontal cortex; RSC, retrosplenial cortex; SC, somatosensory cortex; SN, substantia nigra, STR, striatum; PV, parvalbumin; SOM, somatostatin, and VIP, vasoactive intestinal peptide.

**FIGURE 3:**
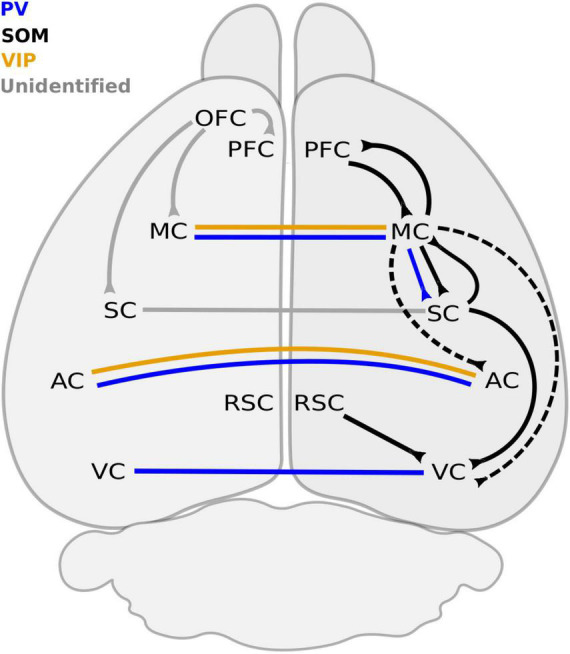
Long-range GABAergic neuron connections between neocortical regions. Ipsilateral connections are mostly dominated by SOM + LRGNs. The PFC sends and receives inhibitory projections from the MC, which also sends inhibitory axons to the SC, AC, and VC. The only reported ipsilateral PV+ connection originates from the MC and terminates in the SC. VC receives SOM + LRGN projections from the MC, SC, and RSC. The molecular identity of LRGNs from the OFC to the PFC, MC and SC remains to be determined. Inhibitory connections between contralateral ACs are PV+ and VIP+, whereas contralateral projections between VCs are PV+ only. The molecular identity of contralateral LRGNs between SCs is unknown. AC, auditory cortex; MC, motor cortex; OFC, orbitofrontal cortex; PFC, prefrontal cortex; RSC, retrosplenial cortex; SC, somatosensory cortex; STR, striatum; VC, visual cortex; PV, parvalbumin; SOM, somatostatin; VIP, vasoactive intestinal peptide. Dashed line refers to connections that remains to be confirmed.

The neocortex is classified as a higher-order center, and it is involved in diverse processes such as perception, control of movement, emotional control, and cognitive function (Mountcastle, 1978; Harris and Shepherd, 2015; Squire et al., 2015). In the canonical circuit, principal pyramidal neurons that are preferentially located in infra-granular layers constitute the main output of the neocortex. As in other cortical regions, LRGNs are present in the neocortex, with their somas located preferentially in layers II/III and V/VI as well as white matter of sensory cortex (Gonchar et al., 1995; Fabri and Manzoni, 2004; Tomioka et al., 2005; Higo et al., 2007, 2009; Rock et al., 2018; Bertero et al., 2021), motor cortex (MC; Tomioka et al., 2005; Rock et al., 2016; Melzer et al., 2017), and associative cortex (Lee et al., 2014; Tomioka et al., 2015). When examined, LRGNs in the neocortex that project to subcortical areas (cortical-fugal) seem to arise from deep layers (Figure 2 and Table 2). In contrast, cortico-cortical LRGNs are more diverse, with soma that span both supra- and infra-pyramidal layers. Given that cortico-cortical projections may target higher or lower-ordered cortical regions (Figure 3 and Table 2), it is possible that this heterogeneity may be linked to bottom-up feedforward vs. top-down feedback functions.

#### Cortico-Fugal Long-Range GABAergic Neurons

##### Somatosensory Cortex

The first cortico-fugal LRGNs discovered were PV neurons from somatosensory cortex (SC) and RSC that project to the caudal striatum (Jinno and Kosaka, 2004), showing scarce but consistent cell body labeling in layer V. Although these retrogradely identified cells have been shown to be GAD immunoreactive, their functional characterization (i.e., inhibition of postsynaptic striatal cells) is lacking.

##### Motor Cortex

Long-range GABAergic neurons in the motor cortex (MC) located in deep layers connect distal subcortical and cortical regions that influence movement behaviors (Tomioka et al., 2005; Rock et al., 2016; Melzer et al., 2017; Bertero et al., 2021). In primary motor cortex (M1), PV + and SOM + GABAergic cells project to the dorsal striatum. M1-LRGNs positive for PV (PV-M1-LRGNs) mainly inhibit D1R-MSNs of the direct pathway, and thus optogenetic activation of their terminals in the striatum increased the duration of immobility bouts (Melzer et al., 2017). On the other hand, M1-LRGNs positive for SOM (SOM-M1–LRGNs) primarily inhibit cholinergic interneurons, and their stimulation induced the opposite effect on locomotion. These results suggest that distinct LRGN subtypes located in the same cortical region can have differing impact on behavior depending on the identity of their targets. Interestingly, LRGNs in the secondary motor cortex (M2) that express SOM (SOM-M2-LRGNs) mainly inhibit striatal D1R-MSNs, and therefore their activation mimics that of PV-M1-LRGNs rather than of SOM-M1-LRGNs (Melzer et al., 2017). Importantly, activation of PV-M1-LRGNs and SOM-M2-LRGNs decrease locomotion by affecting different aspects of movement (i.e., duration of immobility vs. speed of mobility), consistent with the notion that M1 is more associated with motor execution while M2 is more related to motor planning. It will be important to study the precise conditions under which PV-M1-LRGNs and SOM-M2-LRGNs are activated to dissect their specific roles further.

More recently, through viral genetic means, VIP-LRGNs in M1 has been shown to innervate the striatum (Bertero et al., 2021). Their distribution across cortical layers and impact on circuit activity and behavior remains to be determined.

##### Auditory Cortex

In the auditory cortex (AC), LRGNs corresponding to diverse neurochemical classes of cortical GABAergic neurons (e.g., PV, SOM, VIP) have been found, and they project to different subcortical regions. For example, as revealed by viral tracing, SOM + LRGNs projecting to the lateral amygdala (LA) were primarily located in layers V/VI of AC (Bertero et al., 2019), potentially co-mixing with cortico-amygdalar projecting pyramidal neurons (LeDoux et al., 1991) to finetune the emotional significance of auditory signals. Indeed, optogenetic activation of AC-LA LRGNs control the spiking of principal LA neurons (Bertero et al., 2019); however, how they modulate aversive and rewarding behavior has not been investigated. SOM + and PV + LRGNs in deep layers of the AC also project to striatum (Rock et al., 2016; Bertero et al., 2020). Although SOM + LRGNs directly inhibit MSNs, it is unknown whether DR1-MSNs or DR2-MSNs are preferential targets and whether these LRGNs make bifurcating projections to both the amygdala and striatum, as has been reported in subcortical regions and the HPC (Fuchs et al., 2016; Yuan et al., 2017). Intriguingly, the PV + LRGNs exhibit morphology and intrinsic electrophysiological properties similar to PV-INs (Bertero et al., 2020), raising the possibility that individual PV + GABAergic cells may send both local and distal axons.

LRGNs in the auditory cortex can also express VIP, innervating remote areas including the ipsilateral striatum, amygdala and medial geniculate body as well as the superior and inferior colliculus (Bertero et al., 2021). The functional relevance of auditory VIP + LRGNs is not known. VIP + GABAergic cells will likely emerge as a significant population of LRGNs as research in this field continues; for example, in the HPC, VIP + cells projecting to the subiculum are suppressed during locomotion-associated theta rhythms (Francavilla et al., 2018), suggesting that VIP + LRGNs are disengaged during movement to enable the flow of hippocampal-related information. More research is needed to determine whether VIP + LRGNs in the neocortex work similarly.

##### Associative Cortex

mPFC-LRGNs projecting to the NAcc are located in layer II/III and are either PV+ or VIP+ but not SOM+ as revealed in viral tracers in Cre-driver mouse lines (Lee et al., 2014). Although some of the PV-LRGNs display electrophysiological properties similar to PV-INs, not all of them are fast-spiking. Since *in vivo* activation of these inhibitory projections to NAcc elicits avoidance, mPFC-NAcc LRGNs may either directly inhibit D1R-MSNs or inhibit local GABAergic INs to disinhibit D2R-MSNs. However, the specific circuits that drive this avoidance behavior remain to be described. A caveat is the finding that LRGNs in the mPFC also project to other subcortical targets, including dorsal striatum, claustrum and amygdala (Lee et al., 2014). LRGNs also arise from other associative cortical regions. For example, GABAergic cells from the orbitofrontal cortex project to the caudate putamen, with retrogradely labeled soma displaying SOM and PV but not VIP immunoreactivity in mainly infragranular layers (Tomioka et al., 2015). These LRGNs in the orbitofrontal cortex make distributed inhibitory connections, targeting unidentified cell types in other subcortical (e.g., amygdala and substantia nigra) and cortical (e.g., MC, SC, and PFC) regions.

#### Cortico-Cortical Long-Range-GABAergic Neurons

Long-range GABAergic neurons of sensory, motor and associative neocortex have been reported to connect to other neocortical areas in the ipsi- and contralateral hemisphere (Code and Winer, 1985; Buhl and Singer, 1989; Hughes and Peters, 1990; McDonald and Burkhalter, 1993; Gonchar et al., 1995; Fabri and Manzoni, 1996, 2004; Tomioka et al., 2005; Tamamaki and Tomioka, 2010; Rock et al., 2018). Injection of a retrograde tracer in SC labelled LRGNs in M1 and M2 (ipsilateral) with soma localized mainly in layers II/III and V/VI within the same hemisphere (Tomioka et al., 2005). In addition to being positive for GABA, the ipsilaterally projecting MC-SC LRGNs were immunoreactive to diverse markers but show a tendency for shorter and longer projecting LRGNs to express PV and SOM, respectively. Notably, M2 also sends long-range GABAergic afferents to primary and secondary visual cortex, suggesting that most, if not all, primary sensory cortical regions receive movement-related inhibitory feedback (Melzer et al., 2017). Moreover, retrograde tracer injections in M1 revealed reciprocal long-range inhibition from primary and secondary SC but not primary visual cortex (Tomioka et al., 2005). Given that both SC and MC show similar body representations (i.e., homunculus), it remains to be determined whether these SC-MC and MC-SC LRGNs are topographically linked. Although, ipsilateral cortico-cortical inhibitory connections in rodents show similar patterns of connectivity, layer localization and immunoreactivity in other species such as cats (Higo et al., 2007) and monkeys (Tomioka and Rockland, 2007), there is almost no information regarding the cellular targets or the circuit dynamic alterations they exert in brain function and behavior.

Although early research supported the existence of callosal-LRGNs (Code and Winer, 1985; Buhl and Singer, 1989; Hughes and Peters, 1990; Gonchar et al., 1995; Kimura and Baughman, 1997), it was not until 2017 that distal axons from PV-LRGNs in AC, MC and visual cortex to the contralateral counterpart were reported (Rock et al., 2018; Zurita et al., 2018); however, their cellular targets were not identified. Interestingly, callosal AC-LRGNs also send axon collaterals within the AC and inhibit local pyramidal neurons. Although not yet replicated in MC or visual cortex, it is possible that bifurcating callosal-LRGNs in general coordinate activity between bilateral neocortical regions of the same modality. Moreover, there is evidence that LRGNs show similar but not identical electrophysiological properties as their interneuron counterpart. For example, in the AC, callosal PV-LRGNs exhibited a higher expression of a subtype of voltage-sensitive potassium channel (i.e., Kv1.1) than PV-INs and thus were less excitable (Zurita et al., 2018). As callosal VIP-LRGNs was recently found in the AC (Bertero et al., 2021), it will be possible in the near future to determine whether these cells also send collaterals to inhibit GABAergic interneurons and mediate network disinhibition as cortical VIP-INs are known to do in superficial layers (Acsady et al., 1996; Lee et al., 2013; Pi et al., 2013). In order to dissect the circuit motif of callosal long-range inhibition and understand their impact on behavior, more work is needed.

## Long-Range GABAergic Neurons in Development

A clue of LRGN function may come from developmental studies. According to carbocyanine tracing work, corticofugal LRGNs are among the earliest born cells in the murine cortical subplate, being most numerous perinatally and subsequently decreasing in number within the first postnatal week (Boon et al., 2019). Interestingly, contralateral GABAergic projections showed the opposite pattern, gradually increasing during the first postnatal week. Using BrdU birthdating techniques, nNOS + LRGNs in the HPC have also been shown to arise embryonically (Christenson Wick et al., 2019). It is unclear whether these GABA cells can already be distinguished by their long axonal phenotype shortly after birth and what roles they may play during embryonic development. Recent genetic fate mapping studies reveal a subpopulation of highly interconnected early born HUB neurons that may initially shape development of hippocampal networks (Bonifazi et al., 2009) and subsequently mediate long-range GABAergic communication between the HPC and MS in adulthood (Picardo et al., 2011; Villette et al., 2016). More longitudinal studies are needed to determine whether all cortical LRGNs originate from these pioneer HUB neurons and to understand their role in development.

## Neurotransmitter Co-Release in Local and Distant Inhibition

As the list of cortical LRGNs grows, it is becoming clear that these cells often contain neuropeptides that can act as transmitters in addition to GABA. For example, GABAergic projections from the HPC to the MS are SOM+. HPC-amygdala LRGNs can express SOM, NPY and CCK (Lubkemann et al., 2015). Moreover, cortico-cortical LRGNs often co-express SOM, nNOS, or NPY (Tomioka et al., 2005; Higo et al., 2007; Tomioka and Rockland, 2007), whose activity may be enhanced during sleep (Kilduff et al., 2011). The contribution of these bioactive molecules to LRGN function has not been investigated. A possible interaction between GABA and neuropeptide transmission can be garnered from pharmacological work in subcortical regions. In the hypothalamus, GABA agonism *in vivo* can temper the ability of NPY to induce presynaptic secretion of the leutinizing hormone from arcuate nucleus neurons (Horvath et al., 2001). In the basal forebrain, SOM application suppresses GABA release onto cholinergic neurons (Momiyama and Zaborszky, 2006). Moreover, exogenous GABA has been shown to suppress SOM release in *in vitro* cortical preparations (Gemignani et al., 1994). Such findings suggest that neuropeptides and GABA may mutually finetune each other’s influences. However, because neuropeptide secretion from neurons occurs through a pathway distinct from vesicular GABA release, requiring slower dense core vesicles and stronger persistent depolarization (Edwards, 1998; van den Pol, 2012), the physiological conditions that simultaneously recruit GABAergic and peptidergic actions are unknown.

Co-release of multiple neurotransmitters has been shown from projecting subcortical GABAergic neurons. For example, a combination of retrograde tracing and optogenetic electrophysiological recordings show that VTA axons to the habenula and to DG are both GABAergic and glutamatergic (Root et al., 2014; Ntamati and Luscher, 2016). Despite dual release of GABA and glutamate, stimulation of the VTA-DG connection appears to be primarily inhibitory in function, reducing GC firing *in vivo* under anesthesia (Ntamati and Luscher, 2016). Moreover, monosynaptic connections from the supramammillary nucleus of the hypothalamus to GCs and GABAergic interneurons in the DG co-release glutamate and GABA (Hashimotodani et al., 2018). These mixed afferents alone are unable to drive GC firing but can facilitate the excitatory influence of the perforant pathway when co-stimulated. Even at the extensively studied glutamatergic mossy fiber synapse in the HPC, GABA and glutamate co-transmission has been suggested to occur transiently during early postnatal development (Walker et al., 2001; Gutierrez et al., 2003; Beltran et al., 2012), raising the possibility that neurotransmitter co-release may be dynamically regulated.

In the HPC, co-transmission of acetylcholine (ACh) and GABA from optogenetically activated septal fibers occurs *via* different vesicles that are regulated by distinct presynaptic calcium channels and elicits fast GABA-mediated hyperpolarization and slow ACh-mediated depolarization in CA1 pyramidal neurons (Takacs et al., 2018). Interestingly, the GABAergic component of this basal forebrain projection alone is sufficient to suppress sharp wave ripples and epileptiform activity in the HPC. In the frontal cortex, optogenetic activation of cholinergic projections from the basal ganglia triggers co-transmission of GABA and ACh, through separate vesicular pools, resulting in both inhibitory and excitatory postsynaptic influences in local INs, respectively (Saunders et al., 2015).

Neuropeptides do not have to be co-transmitted with GABA to modulate its function. In the hypothalamus, sustained postsynaptic depolarization induces somatodendritic secretion of CCK that subsequently triggers NO production and potentiates presynaptic GABA release (Crosby et al., 2015). Furthermore, in the cortex, SOM can be released following prolonged neural activity (Dao et al., 2019) and has been shown to reduce pyramidal cell excitability (Riedemann and Sutor, 2019) as well as suppress excitatory synaptic drive onto PV-INs (Song et al., 2020). Interestingly, stimulation of PV-INs and SOM-INs has opposing impact on astrocytic calcium signaling in the cortex, with SOM peptidergic activity playing a crucial role (Mariotti et al., 2018). Although these works were focused on cortical INs, the common ability of LRGNs to synthesize SOM as well as other neuropeptides (Paul et al., 2017) posits that peptidergic modulation may also be an important component of LRGN function.

## Long-Range GABAergic Neurons in Brain Function and Physiological Implications

There is a general agreement that behavior not only depends on the activity of one structure but also needs to be coordinated among several brain areas (Friston, 2003; Fries, 2015; Sporns and Betzel, 2016). In this context, the cerebral cortex acts as a hub to multiplex a great variety of processes, ranging from perception to cognition. Understanding how this highly interconnected structure can communicate within different cortical subregions and across subcortical areas is one of the main goals in neuroscience research. In addition to the canonical circuit of excitatory connections, it is becoming increasingly clear that long-range GABAergic inhibition constitutes a new element of the cortical output. Indeed, inhibitory projections may even leave their area of origin alongside long-range excitatory efferents (Rock et al., 2016; Bertero et al., 2019). Consistent with this idea, GABAergic innervations from the somatosensory cortex are intermingled with cortico-fugal glutamatergic ones in the striatum and thalamus (Jinno and Kosaka, 2004). Moreover, layer V pyramidal cells of the RSC have been shown to receive inhibitory projections from CA1 and excitatory inputs from the thalamus (Yamawaki et al., 2019). These examples highlight cortical LRGNs as critical components of feedforward and feedback circuits to counter excitation. As disruption of excitatory-inhibitory balance has been linked to psychiatric disorders like schizophrenia and autism (Marin, 2012; Gonzalez-Burgos et al., 2015), it would not be surprising to learn about contributions of cortical LRGNs in the pathophysiology of these disorders in the near future.

Several recent works support a role of LRGNs in brain function. For example, LEC-CA1 LRGNs have been shown to contribute to sensory processing (Basu et al., 2016). Using *in vivo* calcium imaging, Basu and colleagues demonstrate that the GABAergic axon terminals from the LEC onto CA1 interneurons are active during unimodal sensory stimulation (e.g., air, water, light or tone). Interestingly, during multimodal stimulation, calcium activity of these terminals further increased, suggesting that LEC-CA1 LRGNs integrate multisensory signals to provide contextual information. Indeed, inactivation of these LRGNs impairs learning by changing the specificity and context of formed memories but not memory storage *per se*. At the circuit level, this disinhibitory pathway can shape a form of synaptic plasticity that depends on the timing of inputs; whether this mechanism directly participates in contextual learning remains to be determined. However, it is noteworthy that this study revealed a role of LRGNs in synaptic plasticity that is considered to be a neural correlate of learning and memory (Malenka and Bear, 2004; Whitlock et al., 2006).

In the olfactory system, LRGNs from the cortex and basal forebrain have been suggested to exert inhibitory feedback onto subcortical sensory areas to regulate odor discrimination (Nunez-Parra et al., 2013; Mazo et al., 2020). In this context, LRGNs from the anterior olfactory cortex form synapses with mitral cells, tufted cells and deep-layer GABA INs of the olfactory bulb (OB). Inhibitory projections from the horizontal diagonal band of Broca (HDB) in the basal forebrain modulates OB granule cells, resulting in a net inhibition of both spontaneous and odor-evoked activity in local and output neurons. How these two distinct long-range inhibitory pathways interact to modulate odor perception is awaiting investigation. Notably, HDB-OB LRGNs are under tonic endocannabinoid (eCB) control, showing reduced probability of GABA release in basal conditions that can be alleviated by cannabinoid receptor antagonism (Zhou and Puche, 2021). As eCBs are key retrograde signals in activity-dependent synaptic plasticity, it is likely that OB granule cells can self-regulate the inhibition they receive from these LRGNs to fine-tune odor discrimination. It will be interesting to see whether LRGN inputs from the anterior olfactory cortex can be similarly regulated and whether neuromodulation of non-classical LRGNs can be applied to other brain regions as has been established for the classical subcortical counterparts like the MSNs (Gerfen and Surmeier, 2011; Tritsch and Sabatini, 2012).

Long-range GABAergic neurons in the PFC are anatomically connected to several subcortical areas, but their impact on network function and behavior is only beginning to be revealed. For example, activation of PFC-NAcc LRGNs induces avoidance in a real-time place preference task, suggesting that these inhibitory connections transmit aversive signals and modulate motivational valence (Lee et al., 2014). Interestingly, a more recent study in bioRxiv proposes a previously unknown direct inhibitory connection from the PFC to the HPC that modulates object exploration (Malik et al., 2021). This pathway is composed of diverse LRGNs that specifically inhibit VIP-INs of the CA1 area. As VIP-INs mediate disinhibition of pyramidal neurons, activation of PFC-HPC inhibitory projections leads to a “double disinhibitory long-range motif,” which in turn increases inhibition of hippocampal pyramidal cells and enhances gamma synchrony between the PFC and HPC during exploratory behavior. This is in agreement with the idea that LRGNs coordinate oscillatory activity between distal brain regions (Caputi et al., 2013; Melzer and Monyer, 2020), which facilitates communication and cognition (Engel et al., 2001; Fries, 2015; Abbas et al., 2018; Adaikkan and Tsai, 2020).

**FIGURE 4:**
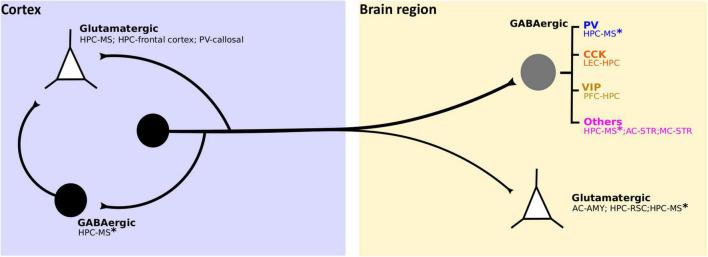
Identified cellular targets of cortical LRGNs. Cortical LRGNs (from HPC or neocortex) project to distal subcortical or cortical areas (yellow box), establishing synapses with different cell subtypes. Some LRGNs (black circle) target excitatory neurons (white triangles) mediating direct long-range inhibition, but the main targets are different subtypes of GABAergic neurons (gray circle). If the postsynaptic targets are PV-INs or Fast Spiking (blue), the final effect of LRGN activity is to increase the output of principal cells in the distal region. By inhibiting dendrite-targeting CCK-INs (orange), LRGNs would potentiate synaptic gain and information flow. Recent non-peer review work reveals potential VIP + targets (dark yellow), which suggests that LRGNs may also act by reducing disinhibition. Moreover, corticofugal LRGNs can target other GABAergic cells (MSNs and diverse INs) and cholinergic INs (pink), whose final impact on circuit function may be multiplicative and affect neuromodulation. Interestingly, some LRGNs like HPC-MS* have diverse targets, thus likely have multiplexed functions. Additionally, LRGNs can locally target inhibitory or excitatory neurons, as HPC-MS LRGNs in DG and callosal PV-LRGNs in the AC, respectively; presumably coordinating both local and distal activity.

PFC-HPC LRGN activation also increases gamma and theta power within the HPC (Malik et al., 2021). Moreover, LRGN projections between the HPC and MS have been shown to play an essential role in hippocampal theta oscillations (Toth et al., 1997; Borhegyi et al., 2004; Jinno et al., 2007; Joshi et al., 2017; Katona et al., 2017; Unal et al., 2018). Such local rhythms likely emerge from the targeting of local GABAergic interneurons (Figure 4), as supported by theoretical models (Buzsaki et al., 2004; Buzsaki and Wang, 2012). Both PV- and SOM-INs are linked to gamma oscillations (Cardin et al., 2009; Sohal et al., 2009; Chen et al., 2017; Veit et al., 2017; Booker et al., 2020) and are phase-locked with theta cycles (Huh et al., 2016). Notably, the impact of local inhibition from the cortex on regional network activity is critically dependent on the target IN subtype. For example, cortical PV-INs are typically rapidly activated by extracortical inputs and exert fast and strong inhibition on principal cell output (Pouille and Scanziani, 2001; Petilla Interneuron Nomenclature Group [PING], Ascoli et al., 2008; Rudy et al., 2011). On the other hand, SOM-INs are generally recruited by local excitation and mainly target dendrites of pyramidal neurons, hence regulating synaptic integration and recurrent microcircuit activity (Silberberg and Markram, 2007; Murayama et al., 2009; Chiu et al., 2013). Moreover, VIP-INs are best known for inhibiting PV- and SOM-INs and thus primarily mediate disinhibition (Acsady et al., 1996; Lee et al., 2013; Pi et al., 2013), setting in place the “double disinhibitory long-range motif” described above (Malik et al., 2021). Taken together, it will be critical to identify the IN subtypes targeted by cortical LRGNs in the future to understand the final impact of distal inhibitory projections from the cortex.

The function of cortical LRGNs is also related to their activation patterns. In the HPC, LRGNs originating from CA1 SO increase their firing during immobility (Katona et al., 2017; Francavilla et al., 2018), but the activity of those in CA1 SR are better coupled to exploration-linked theta oscillations (Jinno et al., 2007), suggesting that SO residing LRGNs act during inactive states whereas SR residing LRGNs work during locomotion (Melzer and Monyer, 2020). Notably, even the immobility-associated LRGN population in SO are heterogeneous, with some but not all cells showing activity that is phase-locked to sharp-wave ripples (Katona et al., 2017; Francavilla et al., 2018). Interestingly, these HPC ripples are linked to memory consolidation in sleep (Buzsaki, 2015; Oliva et al., 2020), potentially in coordination with cortical and subcortical regions (Cox et al., 2020; Norimoto et al., 2020; Skelin et al., 2021). Thus, it is possible that HPC LRGNs may serve to synchronize activity in the HPC as well as in distributed brain regions in memory processing.

Cortical LRGNs in general may function to mediate timed cross-regional dialogue. For example, using viral tracing and optogenetic stimulation, PV + LRGNs have been found to directly connect bilateral cortical regions including the AC, VC and MC (Rock et al., 2018). In AC, these LRGNs are excited by optogenetic stimulation of the auditory thalamus (i.e., medial geniculate body) and robustly inhibit local pyramidal neurons as well. Interestingly, optogenetic activation of undifferentiated callosal projections selectively suppresses and stimulates cortico-cortical and cortico-fugal pyramidal neurons in the AC, respectively (Rock and Apicella, 2015). Although a role for long-range excitation of local INs has been demonstrated in cortico-cortical inhibition, it remains unknown whether and how cortical LRGNs may contribute to the diverging neuronal responses to callosal activity. Regardless, taken together, these findings support the idea that callosal LRGNs may mediate local and interhemispheric feedforward inhibition, synchronizing neural activity in bilateral cortical regions to promote sensory perception and motor response. It is important to note that studies employing vesicular glutamate transporter 2 staining, a classical marker for thalamocortical afferents (Kaneko and Fujiyama, 2002), have suggested that PV + callosal LRGNs (Rock et al., 2018) but not SOM + LRGNs (Tomioka et al., 2005) in MC receive feedforward inputs from the thalamus. Moreover, a recent monosynaptic circuit mapping study suggests that SOM + AC-amygdala LRGNs are excitated by local pyramidal neurons that are innervated by BLA projections (Bertero et al., 2019), thereby forming a cortico-amygdalar loop. Whether synaptic inputs selectively target specific cortical LRGNs remains to be investigated. Such efforts would help us to understand the function of these numerous cortical LRGN types.

## Final Remarks

Although the presence of LRGNs in the cortex challenges the classical view of local inhibition, evidence of their ubiquitous existence and function continues to strengthen. Cortical LRGNs are not simply conventional non-cortical GABAergic projection neurons replicated in the cortex. Canonical subcortical long-range GABAergic projections are more numerous, and even in some cases they emanate from principal cells that express calcium-calmodulin kinase IIα rather than the classical GABA-producing cell markers observed in the cortex (Erondu and Kennedy, 1985; Tepper et al., 2007; Cook et al., 2018). Nevertheless, a model is emerging in which cortical and non-cortical LRGNs have in common a preference to target other GABAergic cells that likely mediate local inhibition.

Cortical LRGNs are not simply cortical INs with long axons, but instead may exhibit distinctive synaptic targeting patterns and kinetic properties. Therefore, although INs and LRGNs share some electrophysiological and molecular similarities, it is presumptuous to think that LRGNs act like their short-range counterparts. As discussed in this review, SOM + LRGNs can deviate from the dendrite-targeting phenotype of SOM-INs in the HPC (Yuan et al., 2017), cortical PV + LRGNs show electrophysiological properties distinct from PV-INs (Lee et al., 2014; Zurita et al., 2018; Yi et al., 2021) and VIP + LRGNs in the HPC target both INs and pyramidal neurons remotely (Francavilla et al., 2018). Moreover, cortical GABAergic cells can possess both short and long axons (Gulyas et al., 2003; Yuan et al., 2017; Rock et al., 2018; Christenson Wick et al., 2019), suggesting that cortical LRGNs may comprise a subpopulation of INs and mediate both local and distant inhibition. It remains to be investigated whether all LRGNs also make short-range synapses. The impact of such dual functionality is unclear but may be beneficial for optimally synchronizing activity between distant regions.

The functional impact of cortical LRGNs on behavior is beginning to be revealed. In this regard, *in vivo* electrophysiological recordings have made great strides. It will be important to determine the circumstances by which these LRGNs are recruited by the network and to assess the causal relationship of their activity to performance in specific tasks. The growing availability of activity-dependent reporters, viral tracers and transgenic mice will facilitate *in vivo* imaging and manipulation of labeled LRGNs throughout the cortex in live animals. In addition, many of the cellular targets of the different LRGNs remain unknown, as well as their pattern of innervation, the properties of the synapses they form and how these synapses are modulated by the great variety of neurochemicals present in the brain. The combination of tracing techniques and high throughput single cell-resolution imaging technology such as light-sheet microscopy will enable the discovery of novel circuits formed by LRGNs as well as propel electrophysiological interrogation of synaptic plasticity and neuromodulation in long-range GABAergic communication. If these long-range inhibitory synapses show activity-dependent plasticity, what is the implication for learning and memory? In addition to discovering new cortical LRGNs, the next steps in research need to focus on advancing our knowledge of the known cortical LRGNs with more detailed study of their function under normal and pathological conditions.

## Author Contributions

JU-P, CM-M, and CQC conceived of the general ideas presented in this review. JU-P and CM-M drove this work to completion, contributing equally to all sections. NS-G and APE contributed to the specific subsections of the written work, provided feedback, and helped with figure construction. CQC supervised the work. All authors made significant contributions to the information content and writing of this final manuscript.

## Conflict of Interest

The authors declare that the research was conducted in the absence of any commercial or financial relationships that could be construed as a potential conflict of interest.

## Publisher’s Note

All claims expressed in this article are solely those of the authors and do not necessarily represent those of their affiliated organizations, or those of the publisher, the editors and the reviewers. Any product that may be evaluated in this article, or claim that may be made by its manufacturer, is not guaranteed or endorsed by the publisher.
